# Antimicrobial Activity of Cultivable Endophytic Fungi Associated with *Hancornia Speciosa* Gomes Bark

**DOI:** 10.2174/1874285801711010179

**Published:** 2017-09-21

**Authors:** Mardonny Bruno de Oliveira Chagas, Irailton Prazeres dos Santos, Luis Claudio Nascimento da Silva, Maria Tereza dos Santos Correia, Janete Magali de Araújo, Marilene da Silva Cavalcanti, Vera Lucia de Menezes Lima

**Affiliations:** 1 Departamento de Bioquímica, Universidade Federal de Pernambuco, Av. Prof. Moraes Rego, S/Nº, Cidade Universitária, Recife, PE, 50670-420, Brazil; 2 Programa de Pós-graduação em Biologia Parasitária, Universidade Ceuma, Rua Josué Montello, São Luís, MA, 65075-120, Brazil; 3 Departamento de Antibióticos, Universidade Federal de Pernambuco, Av. Nelson Chaves, S/N, Cidade Universitária, Recife, PE, 50670-420, Brazil; 4 Departamento de Micologia, Universidade Federal de Pernambuco, Av. Nelson Chaves, Cidade Universitária, S/N, Recife, PE, 50670-420, Brazil

**Keywords:** Fungi diversity, Natural compounds, Semi-solid fermentation, *Aspergillus niger*, *Fusarium solani*, *Proteus mirabilis*

## Abstract

**Introduction:**

In this study, we evaluated the antimicrobial potential of cultivable endophytic fungi associated with *Hancornia speciosa* Gomes stem bark.

**Methods and Materials:**

Plant samples were collected in rainy (July 2010) and dry (January 2011) seasons. In total, 116 endophytic fungi strains were isolated from 90 fragments (64.4% frequency of colonization). Higher fungi frequency was observed in the rainy season (84.4%). The strains were grouped into 14 species; the most frequent were *Phoma cava* (13.8%), *Colletotrichum gloeosporioides* (12.1%), and *Lasiodiplodia theobromae* (11.2%). Fungal diversity was similar in both the seasons. Among the 116 strains, 39 (33.6%) showed antimicrobial activity in preliminary screening. The ten most active isolates were subjected to semi-solid fermentation using rice or corn as substrates. Methanolic extracts were obtained from each fermentation medium and the minimum inhibitory (MIC) and minimum microbicide concentrations (MMC) were determined.

**Results:**

The best antimicrobial results (MIC < 100 µg/mL) were observed for fungi strains grown in rice medium: *Aspergillus niger* FHS061 against *Proteus mirabilis* (MIC = 19 µg/mL) and *Staphylococcus aureus* (MIC = 39 µg/mL). These strains also showed good results when cultivated in corn medium against *P. mirabilis* (MIC = 78 µg/mL).

**Conclusion:**

Thus, the stem bark of *H. speciosa* harbors diverse endophytic fungi with antimicrobial potential.

## 1 INTRODUCTION

Drug resistance is a serious problem worldwide, particularly in hospital environments, contributing to the resurgence of more complex infections and deleterious socioeconomic effects [1-3]. This situation is exacerbated by the increasing number of immuno-compromised patients (elderly subjects with chronic diseases, etc.), continuous flow of travelers and molecular mechanisms of resistance acquisition [4-6]. These factors make essential the search for new and effective antimicrobial agentes. Different sources of natural compounds should be studied in order to exploit the chemical diversity found in nature. Plants can serve as reservoirs for numerous microorganisms classified depending on the locus at which they are found and their functions in the plant. For example, microorganisms known as endophytes colonize plant tissue without causing any apparent symptoms and damages [7, 8].

Endophyte research has stimulated the interest of the scientific community, as these microorganisms represent an important genetic source with the ability to produce metabolites with diverse biotechnological applications [7-9]. Several studies have shown that the colonization and distribution of the endophytic population in plant tissues are regulated by different properties such as environmental factors, microorganism-plant interactions and endophyte community interactions [10, 11]. Due this extensive network of stimuli that regulates the metabolic capacity of endophytic fungi, these microorganisms are excellent targets in the search for compounds with antimicrobial activity, and numerous examples of identified antimicrobials compounds of endophytic fungi have been reported [7].

The pharmacological properties of plants used in folk medicine have been extensively studied, and increasing attention has been given to the biotic components present in these plants [7, 8, 11-13]. *Hancornia speciosa* Gomes (Apocynaceae), a plant native to Brazil and popularly known as mangabeira, is commonly found in the Amazon Rainforest, Cerrado, and Caatinga biomes [14, 15]. This plant has been used to treat various pathologies; some of these properties have been scientifically confirmed such as its antimicrobial [16], antihypertensive [17], antiulcerous [18], antidiabetic [19], anticancer [20], wound healing [21] and anti-inflammatory actions [22, 23]. A recent paper showed that this plant has potential activity against Alzheimer's Disease [24]. However, the microbiota of this plant has not been widely examined, and only one study has evaluated the bacterial community associated with trunk latex [14]. The stem bark of this plant shows antimicrobial activity [16], which was also confirmed by preliminary data from our group (results not published). In this study, we evaluated the antimicrobial activity of endophytic fungi isolated from *H. speciosa* stem bark in two different climatic periods: rainy season (RS) and dry season (DS).

## 2 MATERIAL AND METHODS

Plant samples were collected during the rainy (July 2010) and dry (January 2011) seasons from the Recife coastal region (08º24'334”S, 34º94'384”W), Pernambuco, Brazil. At each sampling period, three samples of stem bark from five healthy specimens of *H. speciosa* were randomly collected, and processed in the Department of Mycology, Federal University of Pernambuco (UFPE).

### 2.1 Isolation of Endophytic Fungi

Initially, healthy samples of stem barks of *H. speciosa* were superficially sterilized to eliminate epiphytic microorganisms. The endophytic fungi were isolated from fragments of stem barks (1 cm^2^) using Petri dishes containing potato dextrose agar (PDA; containing 200 g/L potato, 20 g/L dextrose, and 15 g/L agar, pH 6.0). The whole procedure is comprehensively explained in Santos *et al.* [11].

### 2.2 Identification of Endophytic Fungi

Endophytic fungi from stem barks of *H. speciosa* were identified through macroscopic and microscopic evaluation using the instructions previously reported [25-30]. For this, each colony was grown in PDA, Sabouraud-dextrose agar (SDA), Czapek agar and malt extract agar. Strains showing antimicrobial activity were deposited in the URM Culture Collection (WDCM604) of UFPE. The absolute frequency (f) was estimated as the total number of endophyte isolates, while the relative frequency (rf) was the number of endophytes of each species divided by the total number of endophytic fungi. The rate of colonization was estimated as the total number of fragments of leaves colonized by fungi divided by the total number of fragments used for the isolation of endophytes. The number of isolates in each season was used to determine diversity indices: richness (S), evenness (J′), the Simpson (D′), and Shannon-Wiener (H′) [11].

### 2.3 Test Microorganisms

The antimicrobial activity of endophytic fungi was assayed against test microorganisms provided by the URM Culture Collection and UFPEDA Culture Collection (Department of Antibiotics; UFPE). The following fungi were tested: *Candida albicans* URM**-**5825, *Candida krusei* UFPEDA**-**1002, *Candida tropicalis* URM**-**5871, *Malassezia furfur* URM**-**4220, *Staphylococcus aureus* UFPEDA-02, *Bacillus subtilis* UFPEDA**-**86, *Escherichia coli* UFPEDA**-**224, *Klebsiella pneumoniae* UFPEDA-396, *Pseudomonas aeruginosa* UFPEDA**-**416, and *Proteus mirabilis* UFPEDA**-**767.

### 2.4 Antimicrobial Screening

All endophytic fungi isolated from stem barks of *H. speciosa* were subjected to a preliminary agar diffusion assay as described by Dos Santos *et al.* [12] using Petri dishes previously spread with bacteria (Müller-Hinton agar) and fungi [Sabouraud dextrose agar (SDA) for *Candida* strains and SDA supplemented with 0.5% (SDA+) olive oil for *M. furfur*]. After incubation (37°C for 24 h for bacteria; and at 30°C for 48 h for fungi), the antimicrobial activity was assayed by measuring inhibition diameter zones (IDZ).

### 2.5 Semi-Solid-State Fermentation of Most Active Fungi in Semisolid Medium

The endophytic fungi showing the highest antimicrobial activity were cultured in SDA medium at 28 ± 2°C. After 7 days, five blocks (5 mm) were transferred to Erlenmeyer flasks (1000 mL) containing rice or corn semisolid media (media preparation was performed as previously described [12]. Each fungus was statically cultivated at room temperature (28 ± 2°C). After 30 days, methanolic extracts were prepared as described by Dos Santos *et al.* [12], dissolved in dimethyl sulfoxide (DMSO) and stored at -20°C until use.

### 2.6 Determination of Minimum Inhibitory and Minimum Microbicide Concentrations

Minimum inhibitory concentration (MIC) and minimum microbicide concentration (MMC) were determined by broth microdilution method using specific media (Müller-Hinton agar, SBA or SBA+) as described by Dos Santos *et al.* [12]. Each extract (in 10% DMSO) was tested in concentrations ranging from 9.76 μg/mL to 5000 μg/mL. Each assay was conducted in triplicate.

## 3 STATISTICAL ANALYSIS

All statistical analysis was performed by GraphPad Prism using one-way analysis of variance (ANOVA) and Tukey tests. Data were considered significantly different when p < 0.05. Pearson coefficient (ρ) was used to calculate correlation between the data.

## 4 RESULTS AND DISCUSSION

### 4.1 Endophytic Fungi Isolated from the Stem Bark of *H. Speciosa*

In total, 116 isolates of endophytic fungi were obtained from the 180 fragments analyzed (90 in each period of the year) (Table **1**). These results correspond to a total frequency of colonization (FC) of 64.4%. Regarding the period of the year, 40 isolates were obtained during the dry season (FC of 44.4%), while 76 were obtained during the rainy season (FC of 84.4%). The isolated endophytic fungi were grouped into 14 species: *Trichoderma harzianum* (9.5%), *Phomopsis archeri* (8.7%), *Aspergillus niger* (6.9%), *Penicillium fellutanum* (6.9%), *Nigrospora sphaerica* (6%), and *Fusarium solani* (5.2%). The occurrence of occasional species was also observed in this study, as represented by *Aspergillus flavus*, *Fusarium lateritium*, *Tritirachium oryzae*, *Cladosporium cladosporioides*, and *Marianea elegans*.

Notably, some species of fungi were exclusively isolated during the dry period (*C. cladosporioides* and *M. elegans*) or rainy season (*F. lateritium*, *F. solani*, *P. fellutanum*, *P. archeri*, and *T. oryzae*). The species *P. cava*, *C. gloeosporioides*, and *L. theobromae* showed higher frequencies of colonization. Only a few species were found more frequently compared to the others [31, 32]. The most frequently occurring species are likely those that have a greater affinity to the host plant and perform essential functions such as protection against pathogens [33, 34]. The genera *Aspergillus*, *Fusarium*, *Nigrospora*, *Penicillium*, *Phomopsis*, and *Trichoderma* isolated in this work are frequently reported as endophytes of tropical and subtropical plants [35].

The lowest colonization rates were observed for *C. cladosporioides*, *M. elegans*, and *T. oryzae*. In general, these species can be classified as occasional, as they were represented by a few isolates [32]. In addition, endophytic isolates that did not develop reproductive structures necessary for identification after a certain culture period were also isolated. These isolates were classified as *Mycelia sterilia* [12, 32, 36, 37] (Table **1**).

We observed that the frequency of isolated endophytes in the rainy season was higher than in the dry period. This results may be related to some aspects such as (i) increase of spore dissemination promoted by rain; (ii) facilitation of spore germination by high humidity [38]. Numerous environmental factors are known to influence the frequency of colonization in a plant, including climatic conditions and soil composition at the collection site [10, 11, 38]. Other factors such as fog, rainfall, and dew also play important roles in endophyte diversity, as they can serve as vehicles for fungal spores that penetrate plant tissues and colonize the plant [39].

The diversity indices of isolated fungal populations in each period are shown in Table **2**. The richness (S) values were 1.491 and 1.581 for the rainy season (RS) and dry season (DS), respectively. The RS showed higher values of Shannon-Wiener and evenness indices (H′: 2.506 and J: 0.977) than the DS (H′: 2.097 and J: 0.911). In contrast, the Simpson index was higher during the DS than RS (D = 0.146 and 0.086, respectively). Furthermore, a weak correlation was observed when the number of strains isolated for each species was analyzed (ρ = 0.053); a Sørensen–Dice coefficient of 0.696 was found between both seasons. The richness (S) values were 1.491 and 1.581 for the RS and DS, respectively. The RS showed higher values of Shannon-Wiener and evenness indices (H′: 2.506 and J: 0.977) than the DS (H′: 2.097 and J: 0.911). In contrast, the Simpson index was higher during the DS than the RS (D = 0.146 and 0.086, respectively). These results showed that although different relative frequencies were observed during each season, species diversity was similar.

### 4.2 Screening of Antimicrobial Activity

Endophytic fungi have a higher potential to produce a wide variety of bioactive molecules that belong to various structural classes (such as alkaloids, peptides, steroids, terpenoids, phenols, quinones, and flavonoids). These chemical variety of fungi metabolites is associated with the inhibition of a wide variety of pathogenic microorganisms [7-9, 12, 13].

We examined the antimicrobial action of all 116 endophytic fungi isolated from stem barks of *H. speciosa*. A group of 39 strains (33.6%) displayed antimicrobial activity against Gram-positive bacteria, Gram-negative bacteria, and pathogenic yeasts under the tested conditions. The IDZ values ranged from 11 to 39 mm (Table **3**). In this study, we considered IDZ values smaller than 10 mm as low active, IDZs between 10 and 20 mm moderately active, and IDZs greater than 20 mm as high active [40]. Among the active fungi, 21 were isolated during the RS, while 18 were isolated during the DS. Therefore, the relative frequency of strains with antimicrobial potential was higher in the DS (45%) than in the RS (27.63%) (considering the total number of isolated strains in each season). However, strains isolated in the rainy season had a higher antimicrobial potential because they inhibited the growth of at least two pathogen species.

Regarding the anti-bacterial activity, all strains isolated in the RS showed the capacity to simultaneously inhibit gram-positive and gram-negative bacteria. *Bacillus subtilis* was inhibited by all strains isolated during the RS, with more than 80% presenting IDZ values larger than 20 mm. Similarly, approximately 76% of the endophytic fungi during this period exhibited IDZ larger values than 20 mm against *E. coli*. A significant number of endophytic strains were active against *P. aeruginosa* (67%), *K. pneumoniae* (62%), *S. aureus* (52%), and *P. mirabilis* (43%). In contrast, the bacteria most effectively inhibited by strains isolated in the dry period were *P. aeruginosa* (38.9%), followed by *B. subtilis* (33.3%), *E. coli*, *P. mirabilis*, *S. aureus* (22.2%), and *K. pneumoniae* (11.1%). Finally, only three isolates showed anti-yeast potential. Two strains were effective against *C. albicans* (*A. flavus* FHS012 and *A. niger* FHS059, isolates from the rainy and dry period, respectively) and another strain was active against *M. furfur* (*F. solani* FHS105 from the RS). Other studies have also observed the antimicrobial activity of endophytic fungi from the same genera or species of the strains isolated in our work [12, 31, 32, 41, 42]. The isolates designated as *M. sterilia* were also active against some microorganisms tested, suggesting that antimicrobial potential is independent of the reproductive processes of each isolate, as previously reported [12].

## 4.3 MIC and MBC of the Most Active Endophytic Strains

Eight endophytic fungi isolated from the RS (*A. flavus* FHS012, *A. flavus* FHS018, *A. niger* FHS058, *A. niger* FHS061, *F. solani* FHS105, *F. solani* FHS106, *L. theobromae* FHS070, and *P. fellutanum* FHS089) and two isolated from the DS (*A. niger* FHS059 and *L. theobromae* FHS074) were fermented in semisolid medium. This selection was based on their performance in the antimicrobial screening (IDZ values ≥ 20 mm in diameter against the largest number of pathogenic microorganisms tested). Two semi-solid media (corn or rice) were used for fermentation to obtain crude methanolic extracts, for which the MIC and MMC values were determined. The extracts were dissolved in DMSO, and this solvent did not inhibit microbial growth. Control extracts obtained only from the maize and rice media showed no antimicrobial activity.

All endophytic strains produced antimicrobial compounds in both media tested. The MIC values ranged from 19 μg/mL to 2500 μg/mL (Table **4**). Considering the MIC values of all strans, the most inhibited microrganism was *P. mirabilis* with average MIC of 263.3 μg/mL and 382.6 μg/mL using rice and corn based media, respectively (p < 0.05). In fact, most of the best results (MIC < 100 μg/mL) were observed for extracts obtained from fermentation using rice as a substrate. Regarding the anti-bacterial activity, the most active strains in both medium were: *A. niger* FHS058 (average MIC values of 149.5 μg/mL and 416.5 μg/mL, for rice and corn, respectively), *A. niger* FHS059 (average MIC values of 217.8 μg/mL and 364.3 μg/mL, for rice and corn, respectively), *A. niger* FHS061 (average MIC values of 292.8 μg/mL and 455.5 μg/mL, for rice and corn, respectively). No significant differences were found between their average MIC (p > 0.05). Specifically, when cultivated in rice-based medium, *A. niger* FHS061 showed an MIC of 19 μg/mL towards *P. mirabilis* and 39 μg/mL against *S. aureus*; *A. niger* FHS058 presented MIC values of 78 μg/mL and 39 μg/mL against these same bacteria, respectively. The extract from *F. solani* FHS106 cultivated in rice medium showed an MIC of 78 μg/mL for *K. pneumoniae*.

In corn media, *A. niger* FHS061 had an MIC of 78 μg/mL against *P. mirabilis*. All of these extracts exhibited bacteriostatic activity (MMC/MIC ratio > 4). Some strains exhibited MIC values of 156 μg/mL against *B. subtilis* (FHS058, FHS059, and FHS061 in rice medium), *E. coli* (FHS058 in rice medium), *K. pneumoniae* (FHS106 in rice medium; FHS105 and FHS106 in corn medium), *P. aeroginosa* (FHS058, FHS059, and FHS061 in rice medium; FHS061 in corn medium), and *S. aureus* (FHS058 and FHS059 in corn).

Regarding antifungal action, the strains showed low actitivity against *C. albicans* or *M. furfur.* The best activity against *C. albicans* was observed for the endophytic strains *F. solani* FHS105 (MIC values of 312 μg/mL and in both medium) and *F. solani* FHS106 (MIC values of 312 μg/mL and 625 μg/mL, in corn and rice medium, respectively). These two strains also exhibited the lower MIC values against *M. furfur* (MIC values of 625 μg/mL).

It is well-known that the production of bioactive metabolites depends greatly on the culture medium on which the fungus is grown [8, 43, 44]. In the present study, the best antimicrobial activity was observed when rice was used as a substrate. Recently, our group showed similar results with endophytic fungi isolated from *Indigofera suffruticosa* [12]. In addition to the chemical differences between the two media, interstitial areas were formed between the grains in the rice medium. These areas may promote better substrate utilization in this medium.

### CONCLUSION

Our results demonstrate the diversity of endophytic fungi in *H. speciosa* bark and importance of these microorganisms as promising targets for bioactive compounds with antimicrobial potential. The frequency of colonization of endophytes in *H. speciosa* bark was higher during the rainy season, although diversity was similar in both periods. Crude extracts of the endophytes obtained from fermentation in rice medium showed higher antimicrobial activity. The best results were observed for *A. niger* FHS058, *A. niger* FHS061, and *F. solani* FHS106. Therefore, the isolates described above constitute attractive targets for other studies to optimize the culture conditions for the production of bioactive compounds, as well as their chemical characterization. These compounds may be use directly or as a basis for the synthesis of new agents with antimicrobial activity.

## Figures and Tables

**Table 1:** Endophytic fungi isolated from *H. speciosa* bark in rainy and dry seasons.

**Endophytic fungi**	**RS**		**DS**		**Total**	
**f**	**rf**	**f**	**rf**	**f**	**rf**
*Aspergillus flavus* Link	4	5.3	1	2.5	5	4.3
*Aspergillus niger* Tiegh	5	6.6	3	7.5	8	6.9
*Cladosporium cladosporioides* (Fresen.) G.A. de Vries	-	-	3	7.5	3	2.6
*Colletotrichum gloeosporioides* (Penz.) Penz. & Sacc.	6	7.9	8	20	14	12.1
*Fusarium lateritium* Nees	4	5.3	-	-	4	3.4
*Fusarium solani* (Mart.) Sacc.	6	7.9	-	-	6	5.2
*Lasiodiplodia theobromae* (Pat.) Grif. Maubl.	8	10.5	5	12.5	13	11.2
*Mariannaea elegans* G. Arnaud	-	-	2	5	2	1.7
*Mycelia sterilia*	3	3.9	2	5	5	4.3
*Nigrospora sphaerica* (Sacc.) Mason.	4	5.3	3	7.5	7	6.0
*Penicillium fellutanum* Biourge	8	10.5	-	-	8	6.9
*Phoma cava* Schulzer	6	7.9	10	25	16	13.8
*Phomopsis archeri* B. Sutton.	10	13.1	-	-	10	8.7
*Trichoderma harzianum* Rifai.	8	10.5	3	7.5	11	9.5
*Tritirachium oryzae* (Vincens) de Hoog	4	5.3	-	-	4	3.4
**Total**	**76**		**40**		**116**	

RS: Rainy season; DS: Dry season; f: absolute frequency; rf: relative frequency.

**Table 2:** Diversity, evenness and species richness of endophytic fungi isolated from *H. speciosa* bark in rainy and dry seasons.

Diversity indices	Rainy Season	Dry Season
*Shannon-Wiener index (H′)*	2.506	2.097
*Hmax*	2.565	2.303
*Evenness (J)*	0.977	0.911
*Simpson index (D)*	0.086	0.146
*Richness*	1.491	1.581

**Table 3:** Preliminary antimicrobial screening of endophytic fungi from *H. speciosa* bark isolated in rainy and dry seasons.

**Code**	**Endophytic fungi**	**Season**	**Pathogens**									
			***B. subtilis***	***E. coli***	***K. pneumoniae***	***P. aeruginosa***	***P. mirabilis***	***S. aureus***	***C. albicans***	***C. krusei***	***C. tropicalis***	***M. furfur***
FHS012	*A. flavus*	R.S.	++	++	++	+++	++	++	++	-	-	+
FHS018	*A. flavus*	R.S.	++	+	++	++	++	++	-	-	-	-
FHS019	*A. flavus*	R.S.	++	++	+	++	++	+++	-	-	-	-
FHS061	*A. niger*	R.S.	++	++	++	++	++	+	-	-	-	+
FHS058	*A. niger*	R.S.	++	++	+++	++	++	++	+	+	-	+
FHS042	*C. gloeosporioides*	R.S.	+	-	-	++	-	-	-	-	-	-
FHS051	*F. lateritium*	R.S.	++	++	++	+	-	-	-	-	-	-
FHS052	*F. lateritium*	R.S.	++	++	++	+	-	-	-	-	-	-
FHS101	*F. solani*	R.S.	++	++	+	+	++	++	+	-	-	+
FHS105	*F. solani*	R.S.	++	++	++	++	-	++	+	-	-	++
FHS106	*F. solani*	R.S.	++	++	++	++	+++	++	+	-	-	-
FHS109	*F. solani*	R.S.	++	++	+	++	+	++	+	-	-	+
FHS070	*L. theobromae*	R.S.	++	++	++	++	+	++	-	-	-	+
FHS073	*L. theobromae*	R.S.	++	++	++	++	-	+	-	-	-	-
FHS115	*M. sterilia*	R.S.	+	-	-	+	-	-	-	-	-	-
FHS089	*P. fellutanum*	R.S.	+++	++	++	++	-	++	-	-	-	+
FHS093	*P. fellutanum*	R.S.	++	++	++	++	-	+	-	-	-	-
FHS096	*P. fellutanum*	R.S.	+	++	++	+	-	++	-	-	-	-
FHS005	*P. archeri*	R.S.	++	++	+	-	++	-	-	-	-	-
FHS003	*P. archeri*	R.S.	++	-	-	-	++	-	-	-	-	-
FHS100	*T. oryzae*	R.S.	+	+	-	++	-	-	-	-	-	-
FHS059	*A. niger*	D.S.	++	++	++	++	++	+	++	-	-	-
FHS067	*A. niger*	D.S.	++	+	+	+	-	+	-	-	+	+
FHS041	*C. cladosporioides*	D.S.	-	-	+	++	-	-	-	-	-	-
FHS046	*C. gloeosporioides*	D.S.	+	-	-	++	-	-	-	-	-	-
FHS049	*C. gloeosporioides*	D.S.	-	-	-	+	-	-	-	-	-	-
FHS074	*L. theobromae*	D.S.	++	++	++	++	-	++	-	-	-	-
FHS075	*L. theobromae*	D.S.	++	++	+	++	-	+	-	-	-	-
FHS079	*L. theobromae*	D.S.	++	+	+	++	+	-	-	-	-	-
FHS090	*M. elegans*	D.S.	+	-	-	-	-	-	-	-	-	-
FHS091	*M. elegans*	D.S.	+	-	-	-	+	-	-	-	-	-
FHS114	*M. sterilia*	D.S.	-	++	-	-	-	-	-	-	-	-
FHS116	*M. sterilia*	D.S.	+	-	-	-	-	-	-	-	-	-
FHS031	*N. sphaerica*	D.S.	++	-	-	++	++	++	-	-	-	-
FHS032	*N. sphaerica*	D.S.	+	+	+	+	+	+	-	-	-	-
FHS065	*P. cava*	D.S.	-	-	+	+	++	++	-	-	-	-
FHS066	*P. cava*	D.S.	+	-	+	+	++	++	-	-	-	+
FHS033	*T. harzianum*	D.S.	-	-	-	-	+	-	-	-	-	-
FHS037	*T. harzianum*	D.S.	-	-	-	+	-	-	-	+	-	-

R.S.: Rainy season; D.S.: Dry season; **+**: 20 mm > IDZ 10 mm; **++**: 30 mm > IDZ ≥ 20 mm; **+++**: IDZ ≥ 30 mm; **-**: IDZ < 10 mm.

**Table 4:** Antimicrobial activity of methanolic extracts obtained from semi-solid fermentation of endophytic fungi from *H. speciosa* bark isolated in rainy and dry seasons.

**Code**	**Endophytic fungi**	**Substrate**	**Pathogens**															
			***B. subtilis***		***E. coli***		***K. pneumoniae***		***P. aeruginosa***		***P. mirabilis***		***S. aureus***		***C. albicans***		***M. furfur***	
			**MIC^1^**	**CMM^1^**	**MIC^1^**	**CMM^1^**	**MIC^1^**	**CMM^1^**	**MIC^1^**	**CMM^1^**	**MIC^1^**	**CMM^1^**	**MIC^1^**	**CMM^1^**	**MIC^1^**	**CMM^1^**	**MIC^1^**	**CMM^1^**
FHS012	*A. flavus*	Rice	1250	5000	1250	>5000	1250	5000	1250	2500	312	2500	1250	>5000	2500	>5000	1250	>5000
FHS018	*A. flavus*	Rice	1250	5000	1250	>5000	1250	>5000	1250	2500	312	2500	2500	>5000	2500	>5000	2500	5000
FHS058	*A. niger*	Rice	156	625	156	2500	312	1250	156	1250	78	625	39	312	2500	>5000	2500	5000
FHS059	*A. niger*	Rice	156	1250	625	5000	625	1250	156	2500	39	625	156	625	1250	>5000	2500	>5000
FHS061	*A. niger*	Rice	156	1250	312	5000	625	2500	156	1250	19	625	39	312	2500	5000	2500	>5000
FHS105	*F. solani*	Rice	1250	5000	625	>5000	78	625	625	5000	312	5000	625	1250	312	1250	625	1250
FHS106	*F. solani*	Rice	1250	>5000	625	>5000	156	625	625	5000	312	5000	625	5000	625	1250	625	2500
FHS070	*L. theobromae*	Rice	1250	5000	1250	>5000	1250	2500	312	5000	312	>5000	1250	>5000	625	2500	1250	5000
FHS074	*L. theobromae*	Rice	1250	5000	2500	>5000	1250	5000	625	5000	312	>5000	1250	5000	625	5000	2500	>5000
FHS089	*P. fellutanum*	Rice	1250	>5000	625	5000	1250	>5000	625	>5000	625	5000	1250	>5000	1250	>5000	2500	>5000
FHS012	*A. flavus*	Corn	1250	5000	1250	>5000	1250	>5000	1250	>5000	625	5000	1250	5000	2500	>5000	1250	>5000
FHS018	*A. flavus*	Corn	1250	>5000	2500	>5000	1250	>5000	1250	5000	625	5000	1250	5000	1250	5000	2500	5000
FHS058	*A. niger*	Corn	625	2500	625	2500	625	5000	312	2500	156	1250	156	625	2500	>5000	2500	5000
FHS059	*A. niger*	Corn	625	2500	312	2500	625	2500	312	5000	156	1250	156	1250	2500	5000	1250	5000
FHS061	*A. niger*	Corn	312	1250	312	5000	1250	2500	156	2500	78	625	625	1250	1250	>5000	1250	5000
FHS105	*F. solani*	Corn	625	1250	1250	>5000	156	625	1250	>5000	625	>5000	312	1250	312	1250	2500	>5000
FHS106	*F. solani*	Corn	625	1250	2500	>5000	156	1250	1250	>5000	312	>5000	1250	2500	312	2500	625	1250
FHS070	*L. theobromae*	Corn	2500	5000	1250	5000	2500	5000	625	>5000	312	5000	1250	5000	1250	>5000	1250	2500
FHS074	*L. theobromae*	Corn	2500	5000	1250	5000	2500	>5000	625	>5000	312	>5000	1250	>5000	625	5000	1250	5000
FHS089	*P. fellutanum*	Corn	1250	5000	1250	5000	1250	5000	1250	>5000	625	5000	1250	>5000	2500	>5000	2500	>5000

MIC: minimum inhibitory concentration; MMC: minimum microbicide concentration; ^1^Results are expressed in µg/mL.

## References

[1] Barriere S.L. (2015). Clinical, economic and societal impact of antibiotic resistance.. Expert Opin. Pharmacother..

[2] Martins A., Hunyadi A., Amaral L. (2013). Mechanisms of resistance in bacteria: An evolutionary approach.. Open Microbiol. J..

[3] Tillotson G.S., Zinner S.H. (2017). Burden of antimicrobial resistance in an era of decreasing susceptibility.. Expert Rev. Anti Infect. Ther..

[4] Blair J.M., Webber M.A., Baylay A.J., Ogbolu D.O., Piddock L.J. (2015). Molecular mechanisms of antibiotic resistance.. Nat. Rev. Microbiol..

[5] Millar M.R. (2015). The choice to travel: Health tourists and the spread of antibiotic resistance.. Public Health Ethics.

[6] Hall C.W., Mah T.F. (2017). Molecular mechanisms of biofilm-based antibiotic resistance and tolerance in pathogenic bacteria.. FEMS Microbiol. Rev..

[7] Deshmukh S.K., Verekar S.A., Bhave S.V. (2015). Endophytic fungi: A reservoir of antibacterials.. Front. Microbiol..

[8] Jia M., Chen L., Xin H.L., Zheng C.J., Rahman K., Han T., Qin L.P. (2016). A friendly relationship between endophytic fungi and medicinal plants: A systematic review.. Front. Microbiol..

[9] Gokhale M., Gupta D., Gupta U., Faraz R., Sandhu S.S. (2017). Patents on endophytic fungi.. Recent Pat. Biotechnol..

[10] Higgins K.L., Arnold A.E., Coley P.D., Kursar T.A. (2014). Communities of fungal endophytes in tropical forest grasses: Highly diverse host-and habitat generalists characterized by strong spatial structure.. Fungal Ecol..

[11] Santos I.P., Bezerra J.D., de Souza-Motta C.M., da Silva Cavalcanti M., Menezes Lima V.L. (2015). Endophytic mycobiota from leaves of Indigofera suffruticosa Miller (Fabaceae): The relationship between seasonal change in Atlantic Coastal Forest and tropical dry forest (Caatinga), Brazil.. Afr. J. Microbiol. Res..

[12] Dos Santos I.P., da Silva L.C., da Silva M.V., de Araújo J.M., Cavalcanti Mda.S., Lima V.L. (2015). Antibacterial activity of endophytic fungi from leaves of Indigofera suffruticosa Miller (Fabaceae).. Front. Microbiol..

[13] Negreiros de Carvalho P.L., Silva Ede.O., Chagas-Paula D.A., Hortolan Luiz J.H., Ikegaki M. (2016). Importance and implications of the production of phenolic secondary metabolites by endophytic fungi: A mini-review.. Mini Rev. Med. Chem..

[14] Silva T.F., Coelho M.R., Vollú R.E., de Vasconcelos Goulart F.R., Alviano D.S., Alviano C.S., Seldin L. (2011). Bacterial community associated with the trunk latex of *Hancornia speciosa Gomes* (Apocynaceae) grown in the northeast of Brazil.. Antonie van Leeuwenhoek.

[15] Jimenez H.J., Martins L.S., Montarroyos A.V., Silva Junior J.F., Alzate-Marin A.L., Moraes Filho R.M. (2015). Genetic diversity of the neotropical tree hancornia speciosa gomes in natural populations in Northeastern Brazil.. Genet. Mol. Res..

[16] Costa E.S., Hiruma-Lima C.A., Lima E.O., Sucupira G.C., Bertolin A.O., Lolis S.F., Andrade F.D., Vilegas W., Souza-Brito A.R. (2008). Antimicrobial activity of some medicinal plants of the Cerrado, Brazil.. Phytother. Res..

[17] Ferreira H.C., Serra C.P., Endringer D.C., Lemos V.S., Braga F.C., Cortes S.F. (2007). Endothelium-dependent vasodilation induced by Hancornia speciosa in rat superior mesenteric artery.. Phytomedicine : Int J Phyth Phytopharm.

[18] Moraes Tde.M., Rodrigues C.M., Kushima H., Bauab T.M., Villegas W., Pellizzon C.H., Brito A.R., Hiruma-Lima C.A. (2008). Hancornia speciosa: Indications of gastroprotective, healing and anti-Helicobacter pylori actions.. J. Ethnopharmacol..

[19] Pereira A.C., Pereira A.B., Moreira C.C., Botion L.M., Lemos V.S., Braga F.C., Cortes S.F. (2015). *Hancornia speciosa gomes* (Apocynaceae) as a potential anti-diabetic drug.. J. Ethnopharmacol..

[20] Santos U.P., Campos J.F., Torquato H.F., Paredes-Gamero E.J., Carollo C.A., Estevinho L.M., de Picoli Souza K., Dos Santos E.L. (2016). Antioxidant, antimicrobial and cytotoxic properties as well as the phenolic content of the extract from hancornia speciosa gomes..

[21] Geller F.C., Teixeira M.R., Pereira A.B., Dourado L.P., Souza D.G., Braga F.C., Simões C.M. (2015). Evaluation of the wound healing properties of *Hancornia speciosa* Leaves.. Phytother. Res..

[22] Marinho D.G., Alviano D.S., Matheus M.E., Alviano C.S., Fernandes P.D. (2011). The latex obtained from *Hancornia speciosa gomes* possesses anti-inflammatory activity.. J. Ethnopharmacol..

[23] Torres-Rêgo M., Furtado A.A., Bitencourt M.A., Lima M.C., Andrade R.C., Azevedo E.P., Soares Tda.C., Tomaz J.C., Lopes N.P., da Silva-Júnior A.A., Zucolotto S.M., Fernandes-Pedrosa Mde.F. (2016). Anti-inflammatory activity of aqueous extract and bioactive compounds identified from the fruits of *Hancornia speciosa Gomes* (Apocynaceae).. BMC Complement. Altern. Med..

[24] Penido A.B., De Morais S.M., Ribeiro A.B., Alves D.R., Rodrigues A.L., Dos Santos L.H., de Menezes J.E. (2017). Medicinal plants from northeastern brazil against alzheimer's disease.. Evidence-based complementary and alternative medicine : eCAM.

[25] Morton F.J., Smith G. (1963). The genera scopulariopsis bainier, microascus zukal and doratomyces corda.. Mycological papers.

[26] Ellis M.B. (1971). Dematiaceous Hyphomycetes..

[27] Ellis M.B. (1976). More Dematiaceous Hyphomycetes..

[28] Sutton B.C. (1980). The Coelomycetes: Fungi imperfecti with pycnidia, acervuli and stromata..

[29] Barnett H.L., Hunter B.B. (1987). Illustrated genera of imperfect fungi..

[30] Hanlin R.T. (2000). Illustrated genera of Ascomycetes..

[31] Xing X., Guo S., Fu J. (2010). Biodiversity and distribution of endophytic fungi associated with Panax quinquefolium L. cultivated in a forest reserve.. Symbiosis.

[32] Siqueira V.M., Conti R., Araújo J.M., Souza-Motta C.M. (2011). Endophytic fungi from the medicinal plant Lippia sidoides Cham. and their antimicrobial activity.. Symbiosis.

[33] González-Teuber M. (2016). The defensive role of foliar endophytic fungi for a South American tree.. AoB Plants.

[34] Lugtenberg B.J., Caradus J.R., Johnson L.J. (2016). Fungal endophytes for sustainable crop production.. FEMS Microbiol. Ecol..

[35] Banerjee D. (2011). Endophytic fungal diversity in tropical and subtropical plants.. Res. J. Microbiol..

[36] Su Y.Y., Guo L.D., Hyde K.D. (2010). Response of endophytic fungi of Stipa grandis to experimental plant function group removal in Inner Mongolia steppe.. Fungal Divers..

[37] Mishra V.K., Singh G., Passari A.K., Yadav M.K., Gupta V.K., Singh B.P. (2016). Distribution and antimicrobial potential of endophytic fungi associated with ethnomedicinal plant melastoma malabathricum L.. J. Environ. Biol..

[38] Singh D.K., Sharma V.K., Kumar J., Mishra A., Verma S.K., Sieber T.N., Kharwar R.N. (2017). Diversity of endophytic mycobiota of tropical tree Tectona grandis Linn.f.: Spatiotemporal and tissue type effects.. Sci. Rep..

[39] Arnold A.E., Mejía L.C., Kyllo D., Rojas E.I., Maynard Z., Robbins N., Herre E.A. (2003). Fungal endophytes limit pathogen damage in a tropical tree.. Proc. Natl. Acad. Sci. USA.

[40] Wang F.W., Jiao R.H., Cheng A.B., Tan S.H., Song Y.C. (2007). Antimicrobial potentials of endophytic fungi residing in quercus variabilis and brefeldin a obtained from Cladosporium sp.. World J. Microbiol. Biotechnol..

[41] Hoffman A.M., Mayer S.G., Strobel G.A., Hess W.M., Sovocool G.W., Grange A.H., Harper J.K., Arif A.M., Grant D.M., Kelley-Swift E.G. (2008). Purification, identification and activity of phomodione, a furandione from an endophytic Phoma species.. Phytochemistry.

[42] Chen X.M., Dong H.L., Hu K.X., Sun Z.R., Chen J.A., Guo S.X. (2010). Diversity and antimicrobial and plant-growth-promoting activities of endophytic fungi in Dendrobium loddigesii Rolfe.. J. Plant Growth Regul..

[43] Masi M., Maddau L., Linaldeddu B.T., Scanu B., Evidente A., Cimmino A. (2017). Bioactive metabolites from pathogenic and endophytic fungi of forest trees.. Curr. Med. Chem..

[44] Vasundhara M., Kumar A., Reddy M.S. (2016). Molecular approaches to screen bioactive compounds from endophytic fungi.. Front. Microbiol..

